# Disorder‐Induced Quantum Griffiths Singularity Revealed in an Artificial 2D Superconducting System

**DOI:** 10.1002/advs.201902849

**Published:** 2020-09-18

**Authors:** Xiaowen Han, Yufeng Wu, Hong Xiao, Miao Zhang, Min Gao, Yi Liu, Jian Wang, Tao Hu, Xiaoming Xie, Zengfeng Di

**Affiliations:** ^1^ State Key Laboratory of Functional Materials for Informatics Shanghai Institute of Microsystem and Information Technology Chinese Academy of Sciences 865 Changning Road Shanghai 200050 China; ^2^ Center of Materials Science and Optoelectronics Engineering University of Chinese Academy of Sciences Beijing 100049 China; ^3^ CAS Center for Excellence in Superconducting Electronics (CENSE) Shanghai 200050 China; ^4^ Center for High Pressure Science and Technology Advanced Research Beijing 100094 China; ^5^ International Center for Quantum Materials School of Physics Peking University Beijing 100871 China; ^6^ Collaborative Innovation Center of Quantum Matter Beijing 100871 China; ^7^ CAS Center for Excellence in Topological Quantum Computation University of Chinese Academy of Sciences Beijing 100190 China; ^8^ Beijing Academy of Quantum Information Sciences West Bld. #3, No. 10 Xibeiwang East Rd., Haidian District Beijing 100193 China

**Keywords:** 2D electron gas (2DEG), disorder, quantum Griffiths singularity, quantum phase transition (QPT), vortex pinning energy

## Abstract

Disorder‐induced Griffiths singularity of quantum phase transition (QPT) is a crucial issue in 2D superconductors (2DSC). In a superconducting system, the strength of disorder is found to be associated with the vortex pinning energy, which is closely related to the quantum Griffiths singularity; however, a direct study to elucidate the role of vortex pinning energy on the quantum Griffiths singularity in 2DSC remains to be undertaken. Here, an artificial 2DSC system is designed by randomly depositing superconducting nanoislands on 2Delectron gas (2DEG). Quantum Griffiths singularity is present in a graphene/Pb‐islands‐array hybrid, where the superconducting behavior transits to weakly localized metallic behavior induced by the vertical magnetic field and exhibits critical behavior with a diverging dynamical critical exponent approaching zero temperature. Compared to the study of graphene/Sn‐islands‐array hybrid where the sharp QPT is observed, the vortex pinning energy acquired from the Arrhenius plot analysis is greater in graphene/Pb‐islands‐array hybrid, which may contribute to the presence of the quantum Griffiths singularity. This work may provide a comprehensive interpretation of the QPT in 2DSC.

## Graphical abstract

The disorder‐induced Griffiths type quantum phase transition is observed in 2D graphene/Pb‐islands‐array hybrid at ultralow temperature. Compared to the similar graphene/Sn‐islands‐array hybrid with sharp quantum phase transition, the larger vortex pinning energy of graphene/Pb‐islands‐array hybrid is believed to be responsible for the observed quantum Griffiths singularity.

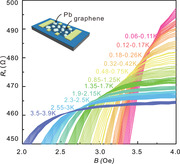

## 1 Introduction

The thermodynamics of 2D superconductors (2DSC) is usually governed by quantum phase transition (QPT) because of the enhanced quantum fluctuation induced by finite‐size effects in a low‐dimensional electron system.^[^ ^1^, ^2^, ^3^, ^4^, ^5^, ^6^, ^7^, ^8^, ^9^, ^10^ ^]^ Superconductor‐insulator/metal transition (SIT/SMT) is a paradigm of QPT, which is the ground state phase transition at zero temperature and is tuned by nonthermal external parameters such as disorder, pressure, and magnetic field.^[^ ^20^ ^]^ The QPT can dominate the thermodynamics up to a relatively high temperature and in fact, the appearance of many unusual superconducting properties results from the proximity of quantum critical points (QCPs), which can be distinguished by a finite‐size scaling (FSS) behavior.^[^ ^20^ ^]^ Quenched disorder (static in time) has a profound influence on the QPT of 2DSC^[^ ^1^ ^]^ and high‐temperature cuprate superconductors that are intrinsically disordered.^[^ ^7^ ^]^

The disorder can suppress global superconductivity in 2DSC by either applying magnetic fields to generate vortices or degrading sample quality.^[^ ^19^ ^]^ The conventional perpendicular magnetic field turned SIT/SMT is usually sharp with a fixed QPT at zero temperature, which has been previously observed in the amorphous and granular 2D superconductors,^[^ ^21^, ^22^, ^23^, ^24^ ^]^ interface superconductor LaTiO_3_/SrTiO_3_,^[^ ^25^ ^]^ under‐doped La_2−_ *_x_*Sr*_x_*CuO_4_^[^ ^26^ ^]^ and graphene/Sn‐islands‐array hybrids.^[^ ^8^ ^]^ While recent experiments instead show a smeared SMT with a divergent dynamical critical exponent (known as the quantum Griffiths singularity) as observed in crystalline superconducting Ga thin film,^[^ ^1^ ^]^ the LaAlO_3_/SrTiO_3_(110) interface,^[^ ^2^ ^]^ monolayer NbSe_2_,^[^ ^3^ ^]^ and ion‐gated ZrNCl film.^[^ ^4^ ^]^ The presence of quantum Griffiths singularity is believed to be caused by a disorder effect in inducing superconducting rare regions (puddles).^[^ ^1^, ^2^, ^3^, ^4^, ^5^, ^6^ ^]^ In a superconducting system, the dynamics of vortices can determine the electromagnetic properties of superconductors^[^ ^7^ ^]^ where the quenched disorder strongly influences the dynamics of systems in pinning the vortex.^[^ ^7^, ^14^ ^]^ Consequently, the strength of disorder is characterized by the vortex pinning energy that is obtained from analysis of the Arrhenius plot.^[^ ^8^, ^15^, ^16^, ^17^, ^18^, ^19^ ^]^ However, previous observations of quantum Griffiths singularity were reported in quite different 2DSC systems,^[^ ^1^, ^2^, ^3^, ^4^, ^5^, ^6^ ^]^ and the relevant vortex pinning energies cannot be compared in parallel between various 2DSC systems. Therefore, a systematic study to elucidate the role of vortex pinning energy on the quantum Griffiths singularity in 2DSC remains to be undertaken. According to our previous work,^[^ ^8^ ^]^ the superconducting puddles‐2DEG hybrid^[^ ^8^, ^9^, ^10^, ^11^, ^12^ ^]^ is an artificial 2D superconducting system, providing a general platform to explore 2D superconductivity with different vortex pinning energies.

In the present study, we deposit Pb nanoislands on single‐crystalline graphene to form a 2D superconducting system consisting of graphene/Pb‐islands‐array hybrid. By applying a vertical magnetic field, the system shows a quantum Griffiths‐type superconductor to metal transition (SMT) in contrast with our previous observation in graphene/Sn‐islands‐array hybrids with different vortex pinning energy,^[^ ^8^ ^]^ where a double quantum critical behavior with a sharp QPT can be realized. Compared to that in graphene/Sn‐islands‐array hybrids,^[^ ^8^ ^]^ the vortex pinning energy^[^ ^13^, ^14^, ^15^, ^16^ ^]^ in the graphene/Pb‐islands‐array hybrid is greater, and is responsible for the quantum Griffiths singularity. These results may improve current understanding of the profound influence of the vortex pinning effect on the QPT in 2DSC.

## 2 Results and Discussion

**Figure 1:**
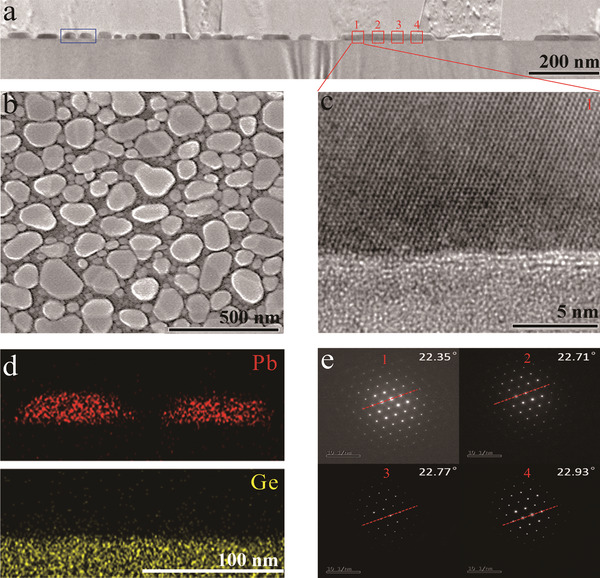
Crystal structure and characterizations of graphene/Pb‐islands‐array hybrids. a) Cross‐sectional TEM image of graphene/Pb‐islands‐array hybrid. b) Plan‐view SEM image of a graphene/Pb‐islands‐array hybrid. c) High‐resolution TEM image of Region 1 marked in Panel (a). d) TEM–EDS elemental maps of the selected blue region in Panel (a) for Pb and Ge. e) SAED patterns collected from different regions of an individual Pb island shown in Panel (a).

Single‐crystalline graphene was synthesized by chemical vapor deposition on an intrinsic Ge (110) substrate (Figure S1, Supporting Information).^[^ ^27^, ^28^ ^]^ Then, 20‐nm‐thick Pb was deposited on single‐crystalline graphene by electron beam evaporation (Experimental Section; Figure S2, Supporting Information). Owing to the poor wettability of graphene^[^ ^8^, ^11^ ^]^ and the low melting point of Pb,^[^ ^29^, ^30^ ^]^ the deposited Pb is prone to being randomly distributed into irregular disconnected nanoislands, as suggested by cross‐sectional transmission electron microscopy (TEM, **Figure** **1**a) and plan‐view scanning electron microscopy (SEM) in Figure 1b. TEM–energy dispersive X‐ray spectroscopy (TEM–EDS, Figure 1d) further proves that Pb nanoislands are completely disconnected from each other. A high‐resolution TEM (HR‐TEM, Figure 1c) image of the selected area of a Pb nanoisland shows that the Pb nanoisland possesses a perfect lattice structure with no distinct grain boundaries. The selected area electron diffraction (SAED) patterns (Figure 1e) collected along an individual Pb nanoisland are nearly identical, evincing the single‐crystalline nature of the Pb nanoisland.^[^ ^31^, ^32^, ^33^ ^]^ Both HR‐TEM image and SAED patterns suggest that the Pb nanoisland possesses a single‐grain structure. As the intrinsic Ge (110) substrate becomes totally insulating below 10 K (Figure S3, Supporting Information), the top single‐crystalline graphene provides an ideal 2DEG platform^[^ ^8^, ^34^ ^]^ that enables 2D coupling between the superconducting Pb nanoislands. Like the superconductor‐metal‐superconductor system acting as a basic Josephson junction,^[^ ^22^, ^35^ ^]^ the Pb nanoislands and the adjacent single‐crystalline graphene constitute Josephson junction arrays.

**Figure 2:**
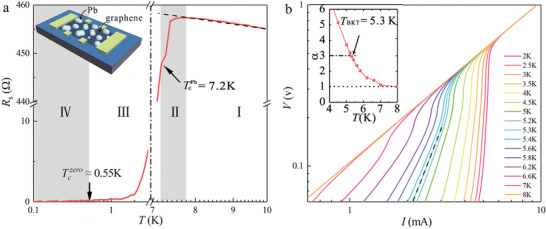
Superconductivity of graphene/Pb‐islands‐array hybrid. a) *R*_s_(*T*) behavior obtained at zero magnetic field. The inset shows a sketch of the Hall bar device. b) Log–log scale plot of the voltage–current (*V*–*I*) curves at various temperatures. The black dashed line represents *V*∝*I* ^3^. The inset shows the extracted power‐law fitting exponent *α* as a function of the temperature. The BKT transition temperature*T*_BKT_ = 5.3K is defined as *α* = 3.

The temperature‐dependent sheet resistance *R*_s_(*T*) at zero magnetic field (**Figure** **2**a and complete data sets are provided in Figure S4a in the Supporting Information) clearly depicts the superconducting transition in the graphene/Pb‐islands‐array hybrid. When cooling from 10 K, the *R*_s_(*T*) curve exhibits semiconductor‐like behavior (d*R*/d*T* < 0, Region I). Since the shunt effect from the intrinsic Ge (110) substrate is absent at temperatures below 10 K, the observed semiconductor‐like behavior is due to the weak localization behavior proposed in 2D metals.^[^ ^36^ ^]^ Then, the system undergoes a two‐step superconducting transition process.^[^ ^9^ ^]^ First, a small decrease in resistance is observed near the critical temperature of bulk Pb ($T_{c}^{\mathrm{Pb}}=7.2\;K$, Region II). As when cooling from $T_{c}^{\mathrm{Pb}}$, the second transition appears due to the Josephson coupling effect and the sheet resistance decreases rapidly in a slide‐like shape to the zero‐resistance transition temperature $T_{c}^{\mathrm{zero}}\approx 0.55K$ (Region III), where *R*_s_(*T*) falls below the resolution limit of our instrument (here, 0.1 Ohms). Region IV corresponds to the true superconducting region with zero resistance. Compared to a graphene/Sn‐islands‐array hybrid,^[^ ^8^ ^]^ the superconducting transition temperature range of a graphene/Pb‐islands‐array hybrid is found to be relatively broad, which may be ascribed to the reduced Josephson coupling effect due to large average spacing of Pb‐islands. The Josephson coupling effect through the 2DEG provided by single‐crystalline graphene is confirmed by comparison experiment in that the graphene/Pb‐islands‐array hybrid becomes insulating when oxygen plasma is utilized to remove the graphene between adjacent Pb nanoislands (Figure S5, Supporting Information). The observed insulating behavior also demonstrates that Pb nanoislands deposited on single‐crystalline graphene are completely disconnected from each other. For a 2D superconducting system, a Berezinskii–Kosterlitz–Thouless (BKT) type transition is expected,^[^ ^37^ ^]^ which interprets the process such that the bound vortex–antivortex pairing breaks into the unbound vortex–antivortex at elevated temperature. The BKT transition temperature (*T*_BKT_) is derived from the *V*–*I* measurements at *V*∝*I* ^3^.^[^ ^1^, ^8^, ^37^ ^]^ In the log–log scale *V*–*I* plot in Figure 2b, a power‐law dependence of *V*∝*I* ^*α*^ behavior is observed, and *T*_BKT_ is 5.3 K where *α* = 3, as indicated by the dash‐dot line in the inset to Figure 2b. The observed BKT transition confirms that the graphene/Pb‐islands‐array hybrid is really a 2D superconducting system.

**Figure 3:**
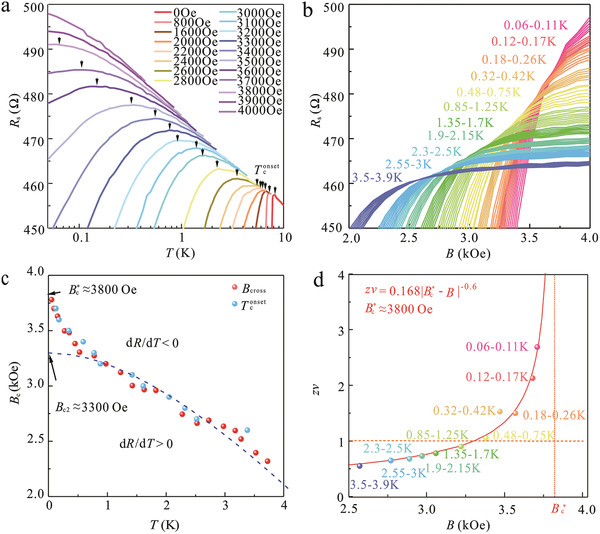
Emergence of the quantum Griffiths singularity. a) *R*_s_(*T*) curves measured under perpendicular magnetic fields varying from 0 to 4000 Oe. The onset superconducting transition temperature *T*_c_^onset^(*B*) is defined as the temperature at which d*R*/d*T* = 0 as marked with black arrows. b) *R*_s_(*B*) as a function of magnetic field at different temperatures varying from 0.06 to 3.9 K. c) A brief *B*–*T* phase diagram yields the boundary of the superconducting state (d*R*/d*T* > 0) and the weakly localized state (d*R*/d*T* < 0). The blue dots represent *T*_c_^onset^(*B*) from the *R*_s_(*T*) curves (a) and the pink dots are *B*_cross_(*T*) representing crossing points of *R*_s_(*B*) curves (b) at each two adjacent temperatures. The *B*_cross_(*T*) data extrapolated to zero temperature gives a critical field $B{\;}_{c}^{*}$ of *≈*3800 Oe. The blue dashed line is the curve fitted using Werthamer–Helfand–Hohenberg (WHH) theory, ln(*T*_0_/*T*)= 1/2Ψ{1/2 + [(*aB* + i*bB*)/(2*πT*/*T*_0_)]} + 1/2Ψ{1/2 + [(*aB* − i*bB*)/(2*πT*/*T*_0_)]} − Ψ(1/2) with Ψ denoting the digamma function, *a* = 2.73 T^−1^,*b* = 0.55 T^−1^, and *T*_0_ = 7.2K, giving a mean‐field upper critical field (*B*_c2_) of about 3300 Oe. d) Exponent *zv* as a function of magnetic field *B*. When approaching the zero‐temperature limit, the *zv* values for each critical region increase rapidly with no signature of saturation. The solid line shows a fitting based on the activated scaling law. Two dashed lines represent the constant values with $B{\;}_{c}^{*}\approx 3800\;\mathrm{Oe}$ and *zv* = 1.

The magnetic‐field‐tuned SMT is essential for 2DSC, and SMT occurs at zero temperature but governs the thermodynamics up to finite temperatures.^[^ ^20^ ^]^ The characteristic of SMT in graphene/Pb‐islands‐array hybrid can be revealed by detailed transport measurements near the QCP. **Figure** **3**a presents the *R*_s_(*T*) curves at different magnetic fields (full data in Figure S4b in the Supporting Information), showing SMT behavior. The *R*_s_(*T*) curves at each field first behave as a weakly localized metal (d*R*/d*T* < 0) and then transit to a superconducting state (d*R*/d*T* > 0) at superconducting onset temperature *T*_c_^onset^(*B*) (indicated by black arrows) as the temperature decreases. And, the system exhibits a complete weak localization behavior once the applied magnetic field exceeds 3800 Oe. *T*_c_^onset^(*B*) shifts monotonically to lower temperatures with increasing magnetic field. To further investigate the SMT behavior near the QCP, magnetoresistances at temperatures from 50 mK to 4 K are measured, as shown in Figure 3b (Figure S6, Supporting Information). It is intriguing to find that the magnetoresistances cross each other at a series of points in a well‐distinguished region. Unlike our previous experimental observations of the graphene/Sn‐islands‐array hybrid^[^ ^8^ ^]^ with two separated crossing points, the appearance of multiple crossing points in graphene/Pb‐islands‐array hybrids is reminiscent of the quantum Griffiths singularity of SMT in Ga film.^[^ ^1^ ^]^ Figure 3c shows a brief *B*–*T* phase diagram consisting of *B*_cross_(*T*) (pink dots, crossing points of two adjacent *R*–*B* curves from Figure 3b) and *T*_c_^onset^(*B*) (blue dots). The tendency of *B*_cross_(*T*) dots is in line with that of *T*_c_^onset^(*B*) dots and both yield the boundary between the superconducting state (d*R*/d*T* > 0) and the weakly localized metallic state (d*R*/d*T* < 0): however, the superconducting boundary exhibits an unusual upturn deviating from the Werthamer–Helfand–Hohenberg (WHH) theory (fitted by the blue dashed line),^[^ ^1^, ^4^, ^38^ ^]^ providing a prediction of mean‐field upper critical field (*B*_c2_) in conventional type‐II superconductors under classical phase transition. At zero temperature, the WHH fitting gives a predicted *B*_c2_ value of about 3300 Oe, which is far below the real critical field $B{\;}_{c}^{*}$ (by extrapolation) of about 3800 Oe. This special upturn is believed to be a consequence of the smeared quantum phase transition (QPT) corresponding to quantum Griffiths singularity.^[^ ^1^, ^2^, ^3^, ^4^, ^5^, ^6^ ^]^

Quantum Griffiths singularity is recognized for its multicrossing behavior and a divergent effective critical exponent *zv* → ∞.^[^ ^1^, ^2^, ^3^, ^4^, ^5^, ^6^ ^]^ To verify the nature of the multiple crossing points, FSS analysis^[^ ^20^ ^]^ is performed. In the FSS analysis, at least three curves are required. So, for the purpose of effective analysis, only the critical transition region formed by more than three adjacent *R*_s_(*B*) curves can be approximated by one quantum “critical” point. Finally, a total of eleven respective “critical” points is obtained in the left‐hand panels of Figures S7–S9 (Supporting Information). According to the FSS law, the resistance near the critical point (*B*_c_,*R*_c_) takes the scaling form expressed thus: *R*_s_(*B*,*T*) = *R*_c_ *F*(|*B* − *B*_c_|*T* ^−1/*zv*^), where *B* is the magnetic field, *B*_c_ is the “critical” field, *R*_c_ is the “critical” resistance and *F*(*x*) represents an arbitrary function of *B* and *T* with *F*(0) = 1. The parameter *v* is the correlation length exponent, *z* represents the dynamical scaling exponent, and *δ* = |*B* − *B*_c_| is the absolute value of distance from the transition. The FSS analysis for each “critical” point is shown in the right‐hand panels of Figures S7–S9 (Supporting Information). The derived *zv* values for each “critical” point are plotted as a function of magnetic field (Figure 3d): it is seen that *zv* values are not constant and diverge as the temperature decreases. In addition, the *zv* values obey an activated scaling law $zv\approx C|B-B_{c}^{*}{|}^{-\upsilon \psi }$ and tend to infinite value when the temperature approaches zero. Here, *C* is a constant coefficient and *υ* ≈ 1.2, *ψ* ≈ 0.5 are the 2D infinite‐randomness critical exponents.^[^ ^1^ ^]^ Our observation is in good agreement with this activated scaling law with *C* = 0.168 and $B{\;}_{c}^{*}\approx 3800\;\mathrm{Oe}$, as shown by the red solid line in Figure 3d. The fitting parameter $B{\;}_{c}^{*}$ agrees with the extrapolation result in Figure 3c, validating the reliability of our fitting process. It is noted that the quantum Griffiths singularity is also observed in another graphene/Pb‐islands‐array hybrid prepared in different batches (Figures S10–S12, Supporting Information), and fitting parameters *C* and $B{\;}_{c}^{*}$ with similar values are obtained (Figure S13, Supporting Information).

The quantum Griffiths singularity in 2D superconductors was first observed in Ga thin film, as a consequence of the formation of rare regions at ultralow temperature due to quenched disorder.^[^ ^1^ ^]^ When approaching zero temperature, rare regions of inhomogeneous superconducting islands gradually emerge and interact via long‐range Josephson coupling to manifest global superconductivity.^[^ ^1^ ^]^ Recent studies show the quantum Griffiths singularity on highly‐crystalline superconductors with weak pinning and disorder.^[^ ^4^ ^]^ In principle, any static disorder will contribute to the increase of pinning^[^ ^7^ ^]^ in a superconducting system, therefore, the strength of disorder is characterized by the vortex pinning energy, which is obtained from analysis of the Arrhenius plot.^[^ ^8^, ^15^, ^16^, ^17^, ^18^, ^19^ ^]^ The Arrhenius plot is often used to study the thermal activated flux flow (TAFF) behavior, where sheet resistance behaves in the form of *R*∝exp ( − *U*(*B*)/*k*_B_ *T*),^[^ ^17^, ^18^, ^19^, ^39^ ^]^ here, *k*_B_ is the Boltzmann's constant and *U*(*B*) is the thermal activation energy, which is the slope of the linear fit in the Arrhenius plot.

**Figure 4:**
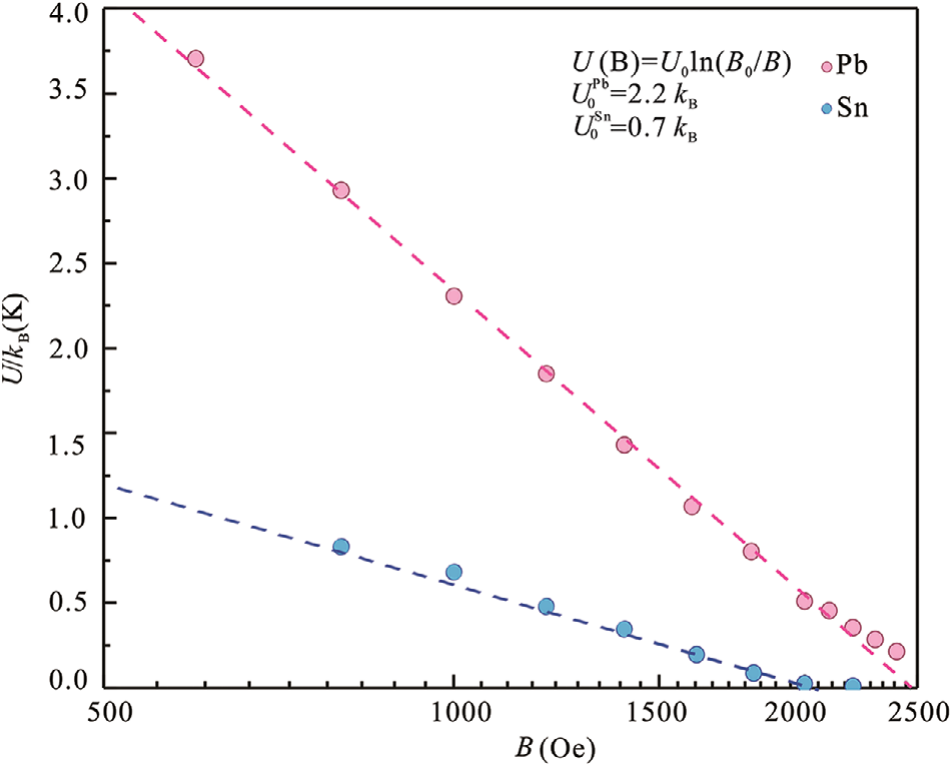
Vortex pinning energy of a graphene/superconductor‐islands‐array hybrid. The magnetic field dependent activation energy extracted from Arrhenius plot analysis fits the thermally‐assisted collective vortex‐creep model in two dimensions expressed as: *U*(*B*) = *U*_0_ln(*B*_0_/*B*), and the slope *U*_0_ corresponds to the vortex pinning energy. The red and blue dots represent the activation energies of a graphene/Pb‐islands‐array hybrid and a graphene/Sn‐islands‐array hybrid (data extracted from Ref. ^[^ ^8^ ^]^), respectively. For parallel comparison purpose, the current densities for both hybrid devices are identical (1 µA mm^−1^) considering the TAFF behavior is extremely sensitive to changes in current density.^[^ ^39^ ^]^

The magnetic field dependent activation energy (Figure S14, Supporting Information) is plotted in **Figure** **4**, which (except in the high magnetic field region) is in consistent with the thermally‐assisted collective vortex‐creep model in a 2D system described by the formula: *U*(*B*) = *U*_0_ln(*B*_0_/*B*),^[^ ^18^, ^19^, ^40^ ^]^ indicating the highly 2D nature of the long range vortex–vortex interaction in the graphene/superconductor‐islands‐array hybrid.^[^ ^41^, ^42^ ^]^ The slope *U*_0_ of the *U*(log *B*) curve in Figure 4 reflects the pinning potential in the superconductor.^[^ ^42^ ^]^ For the graphene/Pb‐islands‐array hybrid analyzed in the present work, the vortex pinning energy is $U_{0}^{\mathrm{Pb}}=2.2k_{B}$: this is more than three times that of the graphene/Sn‐islands‐array hybrid ($U_{0}^{\mathrm{Sn}}=0.7k_{B}$, Figure S15, Supporting Information: data collected from the Supporting Information in Ref. ^[^ ^8^ ^]^). Note that the average vortex pinning energy is $U_{0}^{2}\propto \gamma \xi^{2}$, where *ξ* is the superconducting coherence length and *γ* is the disorder parameter.^[^ ^7^ ^]^ Due to the fact that both graphene/Pb‐islands‐array hybrid and graphene/Sn‐islands‐array hybrid possess a similar superconducting coherence length (Figure S16, Supporting Information), the greater pinning energy may mainly originate from a stronger quenched disorder,^[^ ^7^, ^14^ ^]^ which indicates that strong disorder dominates the dynamic properties of the superconducting system, thus leading to the quantum Griffiths singularity in the graphene/Pb‐islands‐array hybrid. In fact, the assumed stronger disorder in the graphene/Pb‐islands‐array hybrid is verified by the comparison of the surface topographies of Pb and Sn nanoislands on graphene (Figure S17, Supporting Information). According to SEM measurements, the spatial and size distributions of Pb nanoislands are more disordered in the graphene/Pb‐islands‐array hybrid, and there are considerable amounts of tiny scattering islands with only a few nanometers surrounding the core islands, which are rare in the graphene/Sn‐islands‐array hybrid. The presence of tiny scattered Pb islands will introduce additional disorder. Meanwhile, the mixed distribution of different sizes of Pb islands will yield inhomogeneous interisland Josephson coupling energy.^[^ ^9^ ^]^ Therefore, the vortex pinning energies together with the corresponding strength of disorder in the graphene/Pb‐islands‐array hybrid are much greater than that in the graphene/Sn‐islands‐array hybrid. It should be noted that, the strength of the disorder in 2DSC is very complicated, a direct comparison thereof between various 2DSC systems can only be achieved if a simple 2DSC system consisting of ordered superconducting metal nanoislands with variable spacing deposited on graphene can be constructed in the future.

**Figure 5:**
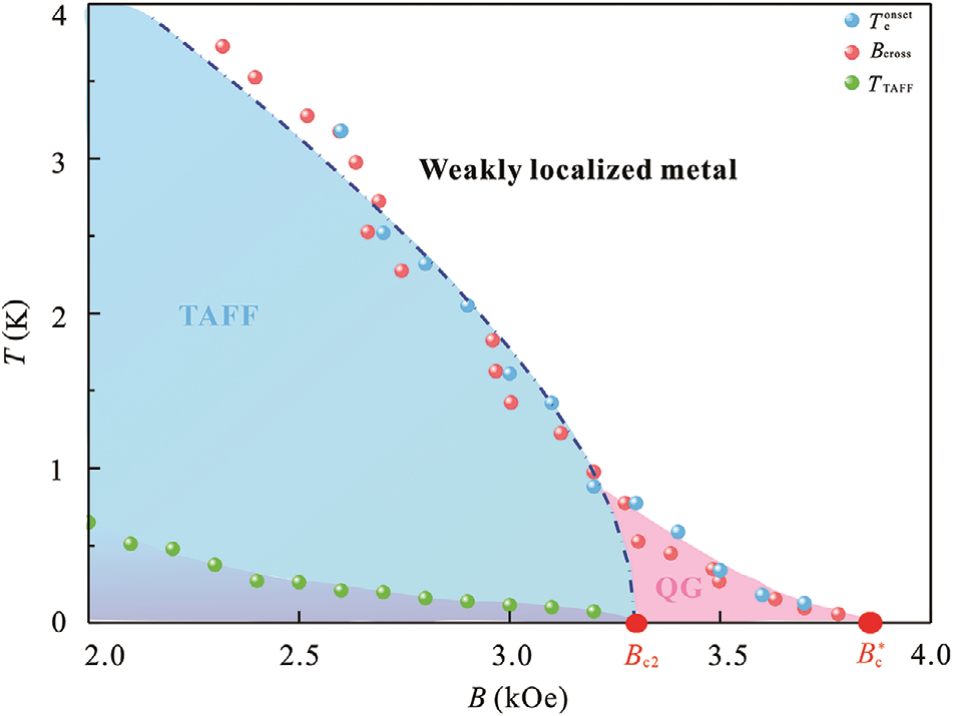
Phase diagram of the graphene/Pb‐islands‐array hybrid. The superconducting dome and the weakly localized metal are separated by the line labelled *T*_c_^onset^(*B*) (blue dots) and *B*_cross_(*T*) (pink dots). The green dots *T*_TAFF_(*B*) shows the boundary of the thermally‐activated flux flow (TAFF) region. The system eventually evolves into a quantum Griffiths (QG) phase when the magnetic field exceeds *B*_c2_ ≈ 3300 Oe (given by the fitting of WHH theory, blue dash‐dot line) to the infinite‐randomness QCP, $B{\;}_{c}^{*}\approx 3800\;\mathrm{Oe}$

With this summary of transport measurement results, a comprehensive *B*–*T* phase diagram for graphene/Pb‐islands‐array hybrid can be established (**Figure** **5**). The boundary of the superconducting region and the weakly localized metal region is characterized by *T*_c_^onset^(*B*) (blue dots) or *B*_cross_(*T*) (pink dots). Most of the boundary can be described by WHH theory (blue dash‐dot line), as is often observed in conventional type‐II superconducting systems. Inside the WHH trace, the green dots *T*_TAFF_(*B*) defined as the detachment of the linear fit in the Arrhenius plot^[^ ^8^, ^10^ ^]^ (marked with black arrows in Figure S14 in the Supporting Information) shows the boundary of the thermally‐activated flux flow (TAFF) region below which quantum fluctuation overtakes thermal fluctuations. It is interesting to note that, in addition to the WHH trace, the boundary exhibits a special upturn, which is never observed in the similar system consisting of a graphene/Sn‐islands‐array hybrid^[^ ^8^ ^]^ (Figure S18, Supporting Information). The special upturn region is found to emerge when the magnetic field exceeds *B*_c2_, but disappears in a magnetic field above $B{\;}_{c}^{*}$. For the upturn region, the superconductivity is dominated by rare regions and the critical exponent *zv* diverging at zero temperature manifests the activated scaling behavior characteristic of the quantum Griffiths singularity. The obtained *B*–*T* phase diagram matches the proposed Griffiths systems,^[^ ^1^, ^2^, ^3^, ^4^ ^]^ indicating that quantum Griffiths singularity truly occurs in graphene/Pb‐islands‐array hybrids as expected.

## 3 Conclusion

In conclusion, the Griffiths‐type QPT has been reproduced in the artificial 2D superconducting graphene/Pb‐islands‐array hybrid. The *B*–*T* phase diagram shows that quantum Griffiths phase persists up to the infinite‐randomness QCP ($B{\;}_{c}^{*}$), which exceeds *B*_c2_ from WHH theory. From the comparative experiments undertaken between Pb and Sn nanoislands on graphene, the observed quantum Griffiths singularity may be attributed to the enhanced vortex pinning energy in graphene/Pb‐islands‐array hybrid. The proposed artificial 2DSC system consisting of single‐crystalline graphene/superconductor‐islands‐array hybrid provides an ideal platform to investigate the influence of disorder on the QPT in 2DSC.

## 4 Experimental Section

Monolayer single‐crystalline graphene was synthesized on the hydrogen‐terminated intrinsic Ge (110) surfaces via an atmospheric pressure chemical vapor deposition technique. The chamber was first evacuated to high vacuum and then aerated by a mixture gas of Ar and H_2_ to atmospheric pressure. Afterwards, the chamber was heated to 916 °C and kept at that temperature during the growth progress with a mixture gas of CH_4_, Ar, and H_2_ for 300 min. Finally, the chamber was quickly cooled to room temperature under the protection of H_2_ and Ar.

The Hall bar device was fabricated as follows: first, a 10 nm Ti/100 nm Au electrode pattern was deposited utilizing a Hall bar stencil mask 1. Then, the graphene (except in the channel region) was etched by oxygen plasma aligned with the stencil mask 2. Finally, a 20‐nm‐thick Pb was deposited by electron beam evaporation on the channel region of the Hall bar device using the designed stencil mask 3.

The temperature and magnetic field dependent resistances were measured by a physical property measurement system (PPMS‐9T, Quantum Design). Ultralow temperature was reached in an He^3^–He^4^ dilution refrigerator (Quantum Design) equipped with a heat capacity cable/RF filter box to eliminate stray RF currents.

## Conflict of Interest

The authors declare no conflict of interest.

## Supporting information

Supporting Information

## References

[1] Y. Xing , H. M. Zhang , H. L. Fu , H. W. Liu , Y. Sun , J. P. Peng , F. Wang , X. Lin , X. C. Ma , Q. K. Xue , J. Wang , X. C. Xie , Science 2015, 350, 542. 26472763 10.1126/science.aaa7154

[2] S. C. Shen , Y. Xing , P. J. Wang , H. W. Liu , H. L. Fu , Y. W. Zhang , L. He , X. C. Xie , X. Lin , J. C. Nie , J. Wang , Phys. Rev. B 2016, 94, 144517.

[3] Y. Xing , R. Zhao , P. J. Shan , F. P. Zheng , Y. W. Zhang , H. L. Fu , Y. Liu , M. L. Tian , C. Y. Xi , H. W. Liu , J. Feng , X. Lin , S. H. Ji , X. Chen , Q. K. Xue , J. Wang , Nano Lett. 2017, 17, 6802. 28967758 10.1021/acs.nanolett.7b03026

[4] Y. Saito , T. Nojima , Y. Iwasa , Nat. Commun. 2018, 9, 778. 29472627 10.1038/s41467-018-03275-z PMC5823914

[5] E. Z. Zhang , J. H. Zhi , Y. C. Zou , Z. F. Ye , L. F. Ai , J. C. Shi , C. Huang , S. S. Liu , Z. H. Lin , X. Y. Zheng , N. Kang , H. Q. Xu , W. Wang , L. He , J. Zou , J. Y. Liu , Z. Q. Mao , F. X. Xiu , Nat. Commun. 2018, 9, 4656. 30405120 10.1038/s41467-018-07123-y PMC6220168

[6] Y. Liu , Z. Q. Wang , P. J. Shan , Y. Tang , C. F. Liu , C. Chen , Y. Xing , Q. Y. Wang , H. W. Liu , X. Lin , X. C. Xie , J. Wang , Nat. Commun. 2019, 10, 3633. 31406114 10.1038/s41467-019-11607-w PMC6690870

[7] G. Blatter , M. V. Feigel'man , V. B. Geshkenbein , A. I. Larkin , V. M. Vinokur , Rev. Mod. Phys. 1994, 66, 1125.

[8] Y. B. Sun , H. Xiao , M. Zhang , Z. Y. Xue , Y. F. Mei , X. M. Xie , T. Hu , Z. F. Di , X. Wang , Nat. Commun. 2018, 9, 2159. 29867112 10.1038/s41467-018-04606-w PMC5986781

[9] S. Eley , S. Gopalakrishnan , P. M. Goldbart , N. Mason , Nat. Phys. 2012, 8, 59.

[10] B. M. Kessler , C. O. Girit , A. Zettl , V. Bouchiat , Phys. Rev. Lett. 2010, 104, 4. 10.1103/PhysRevLett.104.047001 20366731

[11] A. Allain , Z. Han , V. Bouchiat , Nat. Mater. 2012, 11, 590. 22609559 10.1038/nmat3335

[12] Z. Han , A. Allain , H. Arjmandi‐Tash , K. Tikhonov , M. Feigel'man , B. Sacepe , V. Bouchiat , Nat. Phys. 2014, 10, 380.

[13] P. Krueger , Optimisation of Hysteretic Losses in High‐temperature Superconducting Wires, KIT Scientific Publishing, 2014.

[14] B. Karl‐Heinz , J. B. Ketterson , The Physics of Superconductors: Vol. I. Conventional and High‐Tc Superconductors, Springer Science & Business Media, 2012.

[15] O. V. Dobrovolskiy , E. Begun , M. Huth , V. A. Shklovskij , New J. Phys. 2012, 14, 113027.

[16] K. Dinesh , S. Shibnath , K. Sethupathi , M. S. Ramachandra , J. Phys. Commun. 2018, 2, 045015.

[17] Y. Sun , W. H. Zhang , Y. Xing , F. S. Li , Y. F. Zhao , Z. C. Xia , L. L. Wang , X. C. Ma , Q. K. Xue , J. Wang , Sci. Rep. 2015, 4, 6040. 10.1038/srep06040 PMC4129414 25113391

[18] Y. Saito , Y. Kasahara , J. T. Ye , Y. Iwasa , T. Nojima , Science 2015, 350, 409. 26429881 10.1126/science.1259440

[19] A. W. Tsen , B. Hunt , Y. D. Kim , Z. J. Yuan , S. Jia , R. J. Cava , J. Hone , P. Kim , C. R. Dean , A. N. Pasupathy , Nat. Phys. 2016, 12, 208.

[20] S. L. Sondhi , S. M. Girvin , J. P. Carini , D. Shahar , Rev. Mod. Phys. 1997, 69, 315.

[21] A. Yazdani , A. Kapitulnik , Phys. Rev. Lett. 1995, 74, 3037. 10058087 10.1103/PhysRevLett.74.3037

[22] H. S. J. VanderZant , W. J. Elion , L. J. Geerligs , J. E. Mooij , Phys. Rev. B 1996, 54, 10081. 10.1103/physrevb.54.10081 9984747

[23] N. Markovic , C. Christiansen , A. M. Mack , W. H. Huber , A. M. Goldman , Phys. Rev. B 1999, 60, 4320.

[24] N. Markovic , C. Christiansen , A. M. Goldman , Phys. Rev. Lett. 1998, 81, 5217.

[25] J. Biscaras , N. Bergeal , S. Hurand , C. Feuillet‐Palma , A. Rastogi , R. C. Budhani , M. Grilli , S. Caprara , J. Lesueur , Nat. Mater. 2013, 12, 542. 23584144 10.1038/nmat3624

[26] X. Y. Shi , P. V. Lin , D. Popović , G. Logvenov , T. Sasagawa , Nat. Phys. 2014, 10, 437.

[27] J. H. Lee , E. K. Lee , W.‐J. Joo , Y. Jang , B.‐S. Kim , J. Y. Lim , S.‐H. Choi , S. J. Ahn , J. R. Ahn , M.‐H. Park , C.‐W. Yang , B. L. Choi , S.‐W. Hwang , D. Whang , Science 2014, 344, 286. 24700471 10.1126/science.1252268

[28] J. Y. Dai , D. Wang , M. Zhang , T. Niu , A. Li , M. Ye , S. Qiao , G. Ding , X. Xie , Y. Wang , P. K. Chu , Q. Yuan , Z. Di , X. Wang , F. Ding , B. I. Yakobson , Nano Lett. 2016, 16, 3160. 27101021 10.1021/acs.nanolett.6b00486

[29] S. Rolf‐Pissarczyk , J. A. J. Burgess , S. Yan , S. Loth , Phys. Rev. B 2016, 94, 224504.

[30] T. Nishio , T. An , A. Nomura , K. Miyachi , T. Eguchi , H. Sakata , S. Lin , N. Hayashi , N. Nakai , M. Machida , Y. Hasegawa , Phys. Rev. Lett. 2008, 101, 167001. 18999704 10.1103/PhysRevLett.101.167001

[31] X. Z. Xu , Z. H. Zhang , L. Qiu , J. Zhuang , L. Zhang , H. Wang , C. N. Liao , H. Song , R. Qiao , P. Gao , Z. Hu , L. Liao , Z. Liao , D. Yu , E. Wang , F. Ding , H. Peng , K. Liu , Nat. Nanotechnol. 2016, 11, 930. 27501317 10.1038/nnano.2016.132

[32] T. R. Wu , X. F. Zhang , Q. H. Yuan , J. C. Xue , G. Y. Lu , Z. H. Liu , H. S. Wang , H. M. Wang , F. Ding , Q. K. Yu , X. M. Xie , M. H. Jiang , Nat. Mater. 2016, 15, 43. 26595118 10.1038/nmat4477

[33] M. Huang , M. Biswal , H. J. Park , S. Jin , D. Qu , S. Hong , Z. Zhu , L. Qiu , D. Luo , X. Liu , Z. Yang , Z. Liu , Y. Huang , H. Lim , W. J. Yoo , F. Ding , Y. Wang , Z. Lee , R. S. Ruoff , ACS Nano 2018, 12, 6117. 29790339 10.1021/acsnano.8b02444

[34] A. K. Geim , K. S. Novoselov , Nat. Mater. 2007, 6, 183. 17330084 10.1038/nmat1849

[35] H. S. J. Vanderzant , F. C. Fritschy , W. J. Elion , L. J. Geerligs , J. E. Mooij , Phys. Rev. Lett. 1992, 69, 2971. 10046688 10.1103/PhysRevLett.69.2971

[36] L. Vandendries , C. Vanhaesendonck , Y. Bruynseraede , G. Deutscher , Phys. Rev. Lett. 1981, 46, 565.

[37] N. Reyren , S. Thiel , A. D. Caviglia , L. F. Kourkoutis , G. Hammerl , C. Richter , C. W. Schneider , T. Kopp , A.‐S. Rüetschi , D. Jaccard , M. Gabay , D. A. Muller , J.‐M. Triscone , J. Mannhart , Science 2007, 317, 1196. 17673621 10.1126/science.1146006

[38] N. R. Werthame , E. Helfand , P. C. Hohenber , Phys. Rev. 1966, 147, 295.

[39] I. Tamir , A. Benyamini , E. J. Telford , F. Gorniaczyk , A. Doron , T. Levinson , D. Wang , Sci. Adv. 2019, 5, eaau3826. 30899781 10.1126/sciadv.aau3826 PMC6420316

[40] M. V. Feigelman , V. B. Geshkenbein , A. I. Larkin , Phys. C 1990, 167, 177.

[41] O. Brunner , L. Antognazza , J. Triscone , L. Miéville , O. Fischer , Phys. Rev. Lett. 1991, 67, 1354. 10044124 10.1103/PhysRevLett.67.1354

[42] G. Karapetrov , A. Belkin , V. Novosad , M. Iavarone , J. Fedor , J. E. Pearson , A. Petrean‐Troncalli , IEEE Trans. Appl. Supercond. 2009, 19, 3471.

