# Supplementary material for: Disorder‐Induced Quantum Griffiths Singularity Revealed in an Artificial 2D Superconducting System
Source: Adv Sci (Weinh). 2020 Sep 18;7(20):1902849. doi: 10.1002/advs.201902849 (PMC7578859; doi:10.1002/advs.201902849)
Supplement: Supplementary file 1 — Supporting Information [file ADVS-7-1902849-s001.pdf]

© 2020 Wiley-VCH GmbH

ADVANCED SCIENCE
Open Access

Supporting Information

for *Adv. Sci.*, DOI: 10.1002/advs.201902849

Disorder-induced Quantum Griffiths Singularity Revealed in an Artificial Two-dimensional Superconducting System

Xiaowen Han, Yufeng Wu, Hong Xiao, Miao Zhang, Min Gao, Yi Liu, Jian Wang, Tao Hu, Xiaoming Xie, and Zengfeng Di**

Supporting Information

Disorder-induced quantum Griffiths singularity revealed in an artificial two-dimensional superconducting system

Xiaowen Han[#], Yufeng Wu[#], Hong Xiao, Miao Zhang, Min Gao, Yi Liu, Jian Wang, Tao Hu[*], Xiaoming Xie, and Zengfeng Di[*]

*Corresponding author. Email: hutao@baqis.ac.cn; zfdi@mail.sim.ac.cn

This PDF file include:

Supporting Text

Supporting Figures S1 to S18

References

Supporting Text

1. Device fabrication

Monolayer single-crystalline graphene was synthesized on the hydrogen-terminated intrinsic Ge (110) surfaces via an atmospheric pressure chemical vapour deposition (CVD). The chamber was first evacuated to high vacuum and then aerated by a mixture gas of Ar and H_2 to atmospheric pressure. Afterwards, the chamber was heated to 916 ^{o}C and kept at that temperature during the growth progress with a mixture gas of CH_4, Ar, and H_2 for 300 min. Finally, the chamber was quickly cooled to room temperature under the protection of H_2 and Ar.

The Hall bar device was fabricated as follows: firstly, 10 nm Ti/100 nm Au electrode pattern was deposited utilizing a Hall bar stencil mask 1. Then, the graphene except the channel region was etched by oxygen plasma aligned with the stencil mask 2. Finally, 20-nm-thick Pb was deposited by electron beam evaporation very slowly to the channel region of the Hall bar device using the designed stencil mask 3.

2. **Transport measurements.**

The temperature and magnetic field dependent resistance was measured by a physical property measurement system (PPMS-9T, Quantum Design). Ultralow temperature was reached in an He^3 -He^4 dilution refrigerator (Quantum Design) equipped with a heat capacity cable/RF filter box to eliminate stray RF currents.

3. **The estimation of coherence length.**

The superconducting coherence length is estimated by the standard linearized Ginzburg–Landau (GL) theory, [1] i.e., $H_{c2,\perp} = \frac{\emptyset_0}{2\pi\xi_{\mathrm{GL}}(0)^2}(1 - \frac{T}{T_c})$, where $\xi_{\mathrm{GL}}(0)$ is the zero-temperature GL in-plane coherence length and $\emptyset_0 = 2.07 \times 10^{-15}$ Wb is the magnetic flux quantum. $H_{c2,\perp}$ is defined as the 90% resistance of the normal state just above the onset. From the slope of the linear fitting, we can derive that the zero-temperature GL in-plane coherence length for graphene/Pb-islands-array hybrid and graphene/Sn-islands-array hybrid are ~37 nm and ~41 nm. The data near T_c diverges from the linear fit because the 2D superconductivity corresponding to the second superconducting transition region emerges.

4. **Arrhenius plot analysis and TAFF region.**

The Arrhenius plot is often used to study the thermally activated flux flow (TAFF) behavior, where resistivity at different magnetic field is plotted as the logarithm of resistivity against the inverse temperature. In the TAFF region, thermal fluctuations activate the bound vortices and the motion of such vortices driven by the Lorentz force gives the sheet resistance behaving in the form of $R \propto \exp(-U(B)/k_\mathrm{B}T)$, where k_B is the Boltzmann's constant and $U(B)$ is the thermally activated energy, which will be a linear trace in the Arrhenius plot. As guided to eye, when cooling down the sample, the resistance first drops in a linear trace (black dash lines) corresponding to the TAFF behavior. The characteristic temperature of TAFF region ($T_\mathrm{TAFF}(B)$) is defined as the deviation of the dash linear fit. The slope of the linear fit represents the activation energy.

Supporting Figure

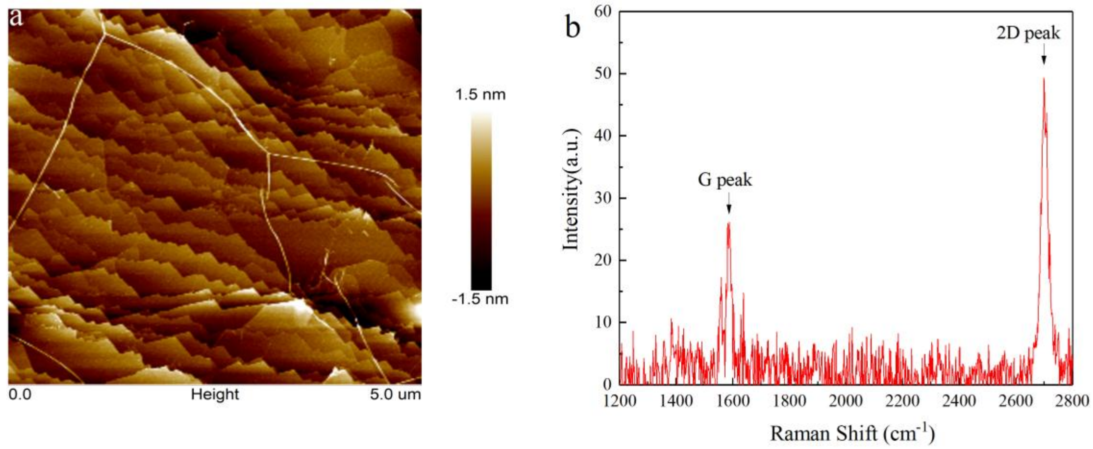

Figure S1. Characterization of single-crystalline monolayer graphene. a) AFM image of the single-crystalline monolayer graphene grown on the intrinsic Ge (110) wafer. b) Raman spectra shows a sharp 2D peak and G peak at 2680 cm^{-1}, 1580 cm^{-1} separately. The high ratio of 2D/G being ~1.7 and the absence of D peak near 1300 cm^{-1} indicate that the single-crystalline monolayer graphene is obtained.

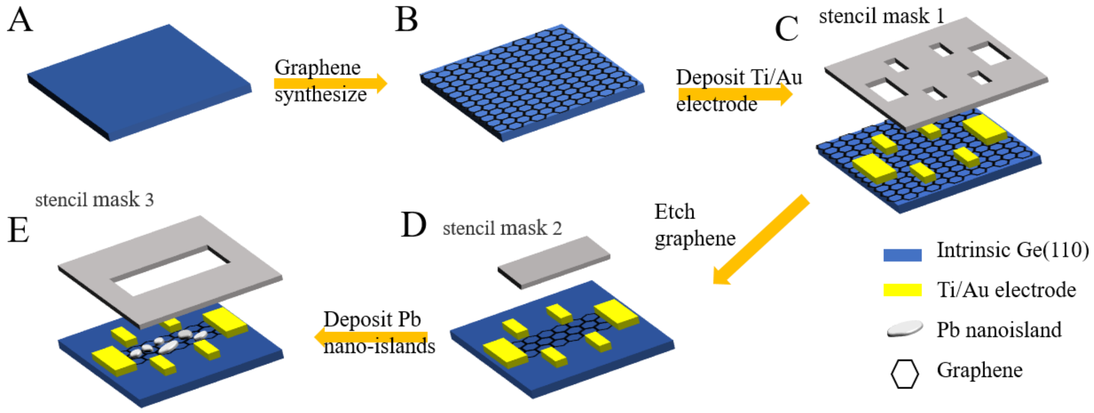

Figure S2. Sketch illustration of the device fabrication. A-B) Single-crystalline monolayer graphene was synthesized on intrinsic Ge(110) substrate. B-C) 10 nm Ti/100 nm Au electrode was deposited on the graphene. C-D) The graphene except the channel was etched by oxygen plasma. D-E) 20 nm Pb was slowly deposited on the graphene.

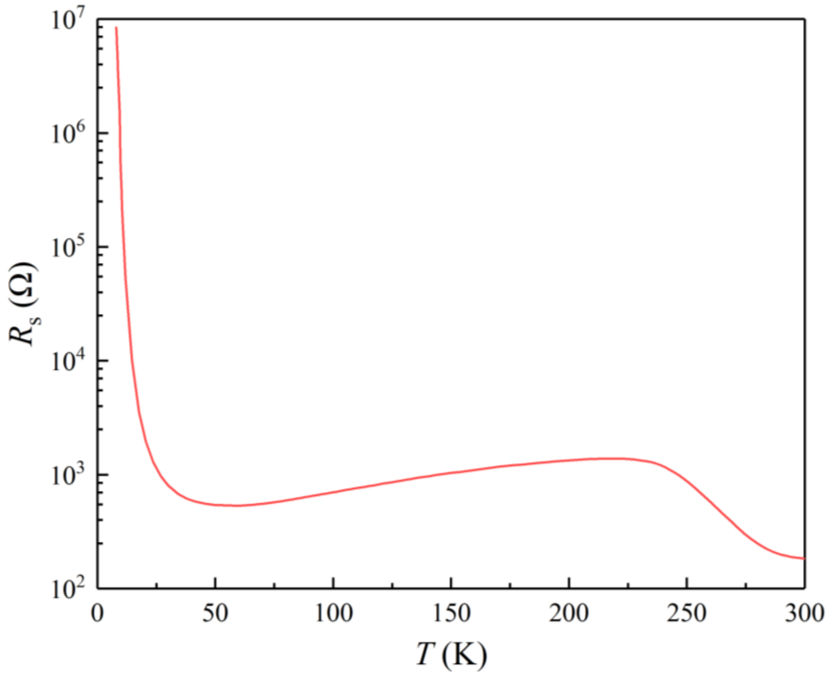

Figure S3. Transport measurement on intrinsic Ge(110) substrate. $R_s(T)$ measurement shows that intrinsic Ge(110) substrate becomes insulating at the temperature below 10K.

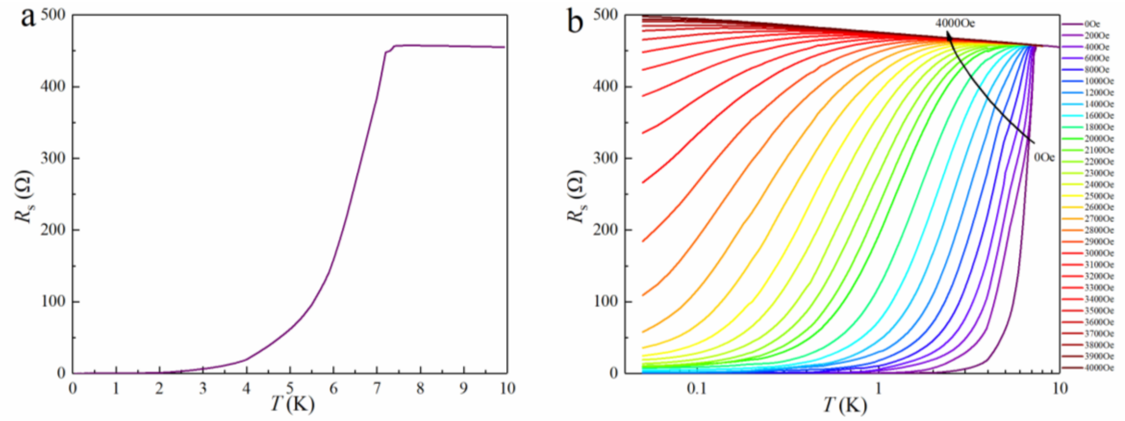

Figure S4. Full data of $R_s(T)$ curves. The $R_s(T)$ curves are measured at (a) zero magnetic field and (b) different vertical magnetic fields ranging in 200 Oe steps from 0 Oe to 2000 Oe and in 100 Oe steps from 2100 Oe to 4000 Oe.

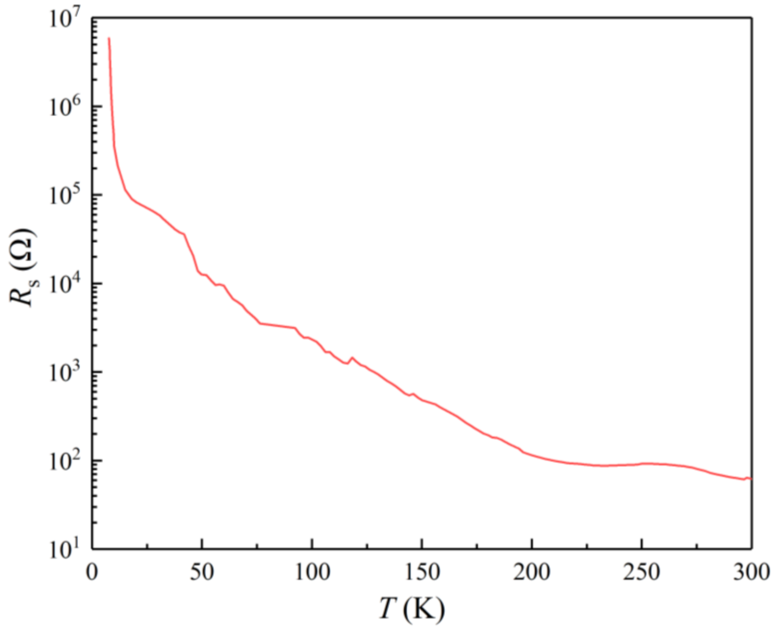

Figure S5. Transport measurement of graphene/Pb-islands-array hybrid after oxygen plasma etching. $R_s(T)$ behavior shows that graphene/Pb-islands-array hybrid becomes insulating when oxygen plasma is utilized to remove the graphene between adjacent Pb nano-islands.

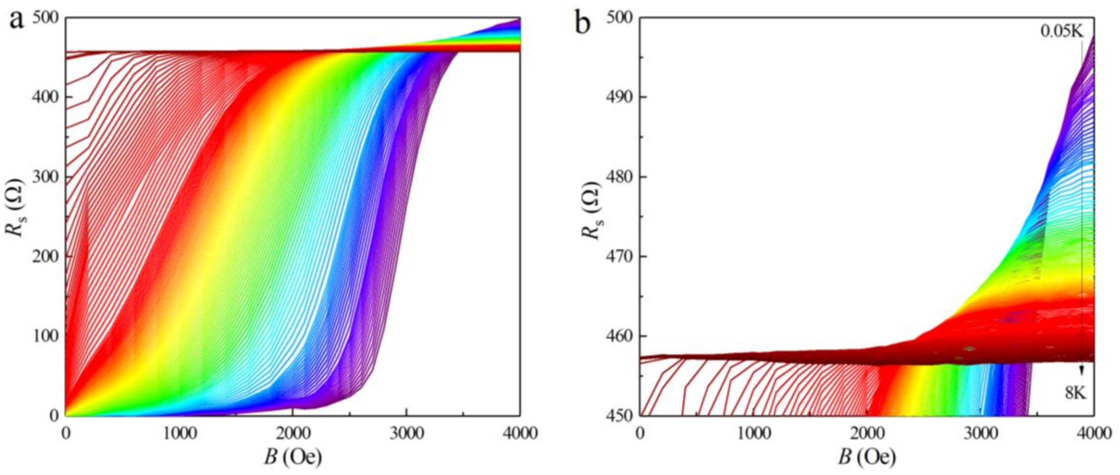

Figure S6. Full data of Magnetoresistance isotherms. $R_s(B)$ curves are measured in 10 mK steps from 50 mK to 200 mK, 20 mK steps from 220 mK to 0.5 K, 50 mK steps from 0.55 K to 4 K and 0.1 K steps from 4.1 K to 8 K. **a** The entire data. **b** Detailed image of the multi-crossing area.

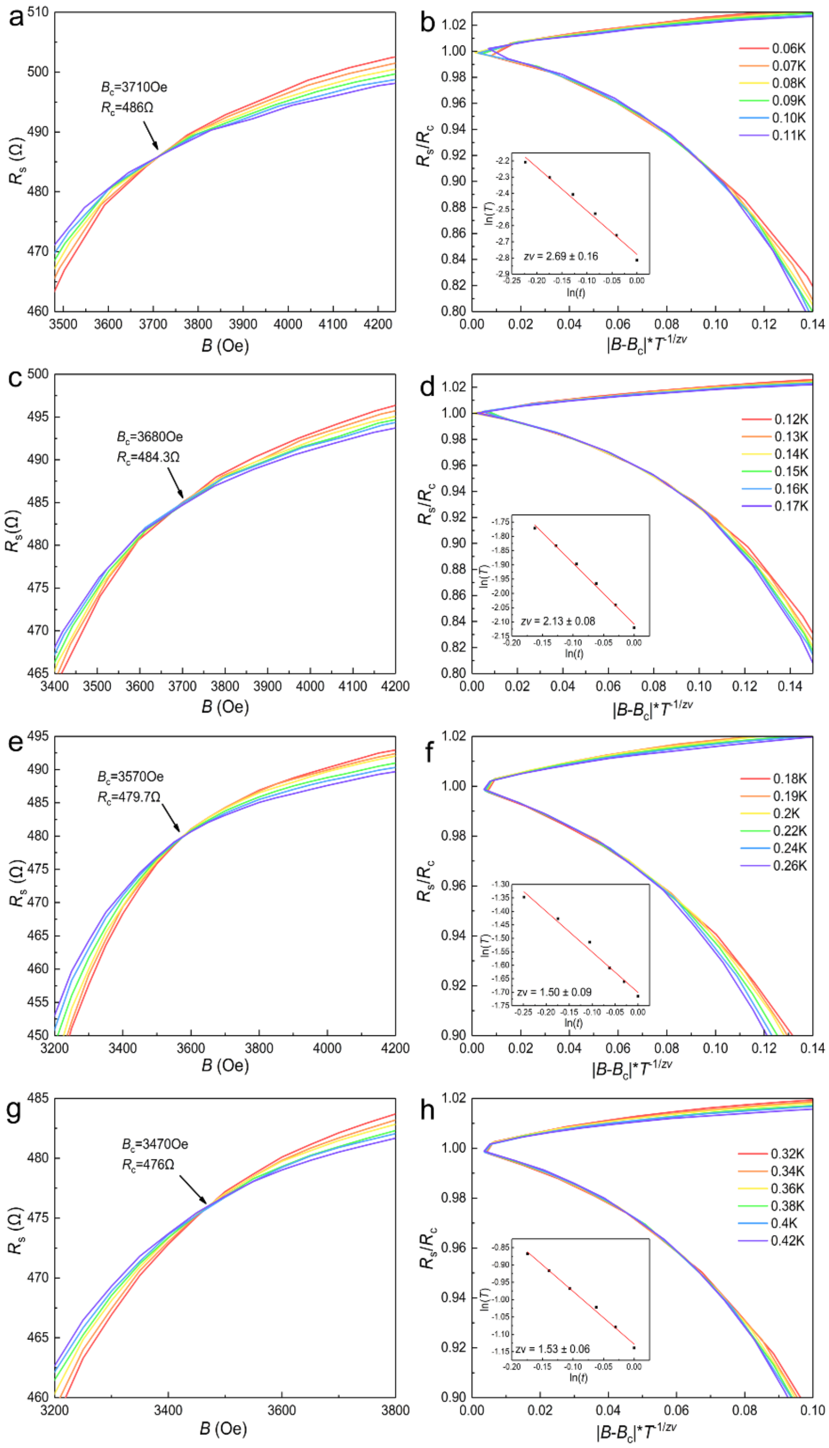

Figure S7. Finite-size scaling analysis for different "critical" points from 0.06 K to 0.42 K. a, c, e, g) $R_s(B)$ characteristics of different temperatures crossover at "critical" point (B_c, R_c). b, d, f, h) Normalized R_s as a function of the scaling variable $\left|B - B_c\right|(T / T_0)^{-1/zv}$. Inset shows the relationship between the temperature and the scaling parameter t as $t = T^{-1/zv}$ in a log-log scale plot and the critical exponent zv is

derived from the linear fitting.

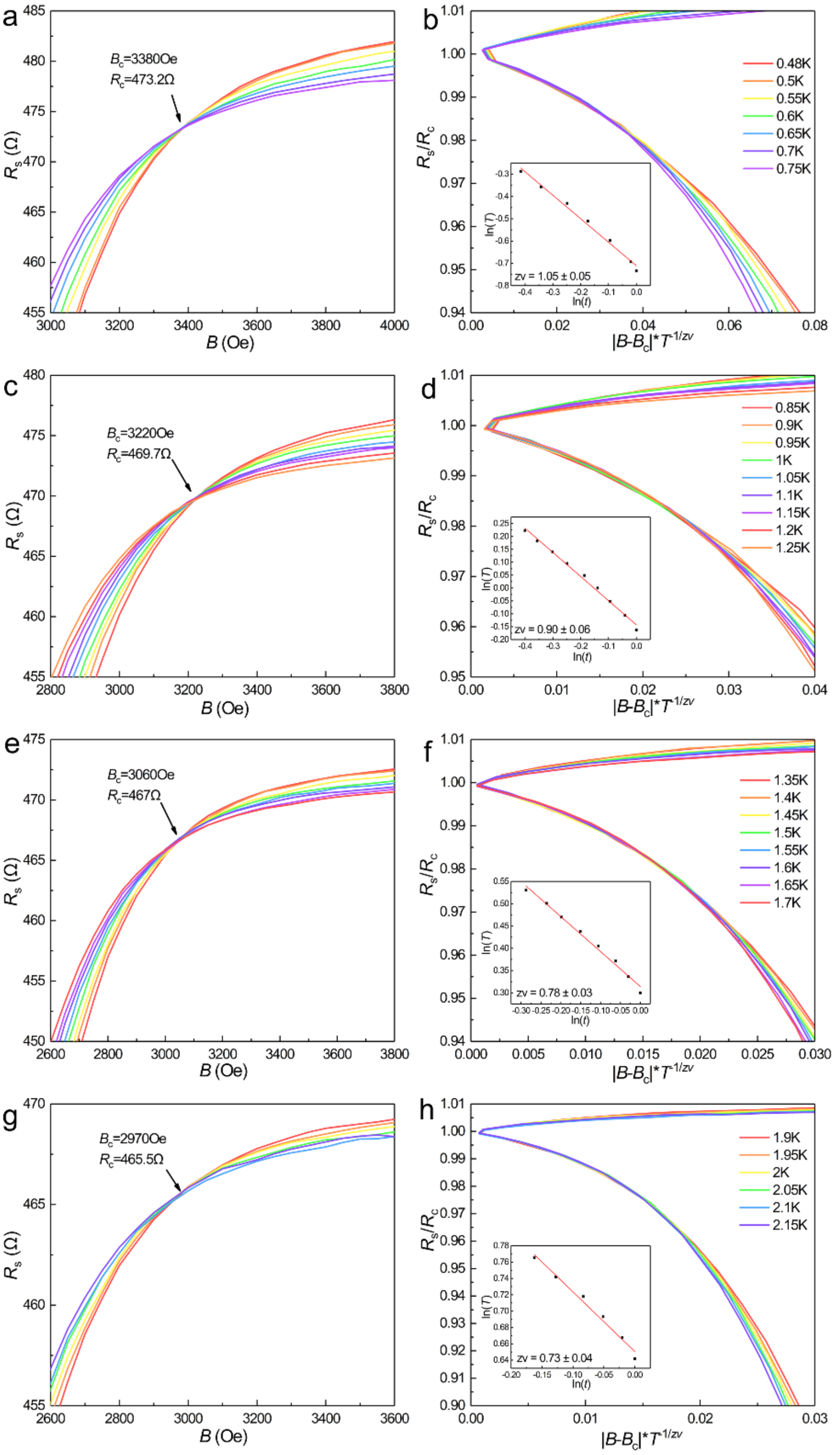

Figure S8. Finite-size scaling analysis for different "critical" points from 0.48 K to 2.15 K. a, c, e, g) $R_s(B)$ characteristics of different temperatures crossover at "critical" point (B_c, R_c). b, d, f, h) Normalized R_s as a function of the scaling variable $\left|B - B_c\right|(T / T_0)^{-1/zv}$. Inset shows the relationship between the temperature and the scaling parameter t as $t = T^{-1/zv}$ in a log-log scale plot and the critical exponent zv is derived

from the linear fitting.

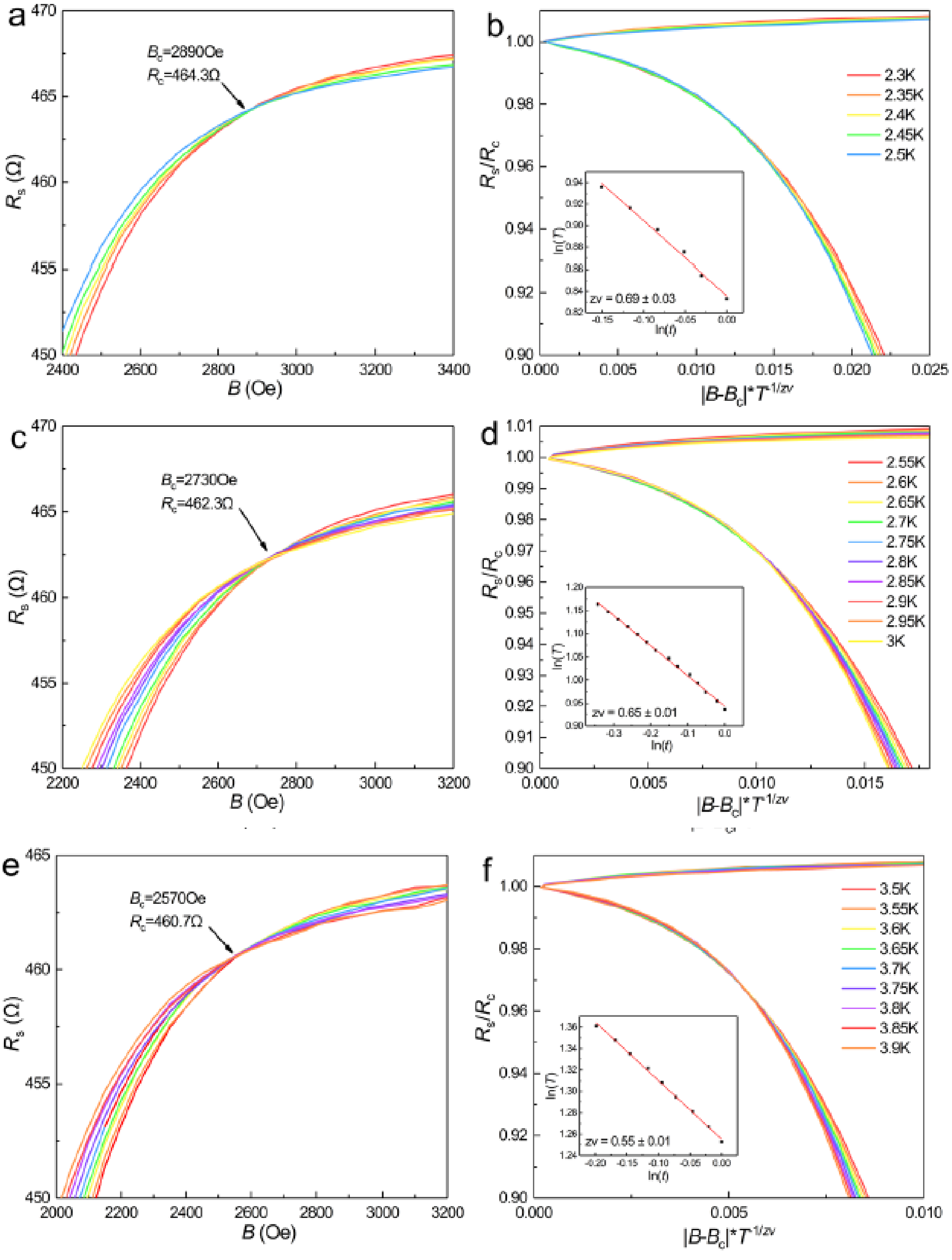

Figure S9. Finite-size scaling analysis for different "critical" points from 2.3 K to 3.9 K. a, c, e) $R_s(B)$ characteristics of different temperatures crossover at "critical" point (B_c, R_c). b, d, f) Normalized R_s as a function of the scaling variable $|B - B_c|(T / T_0)^{-1/zv}$. Inset shows the relationship between the temperature and the scaling parameter t as $t = T^{-1/zv}$ in a log-log scale plot and the critical exponent zv is derived from the linear

fitting.

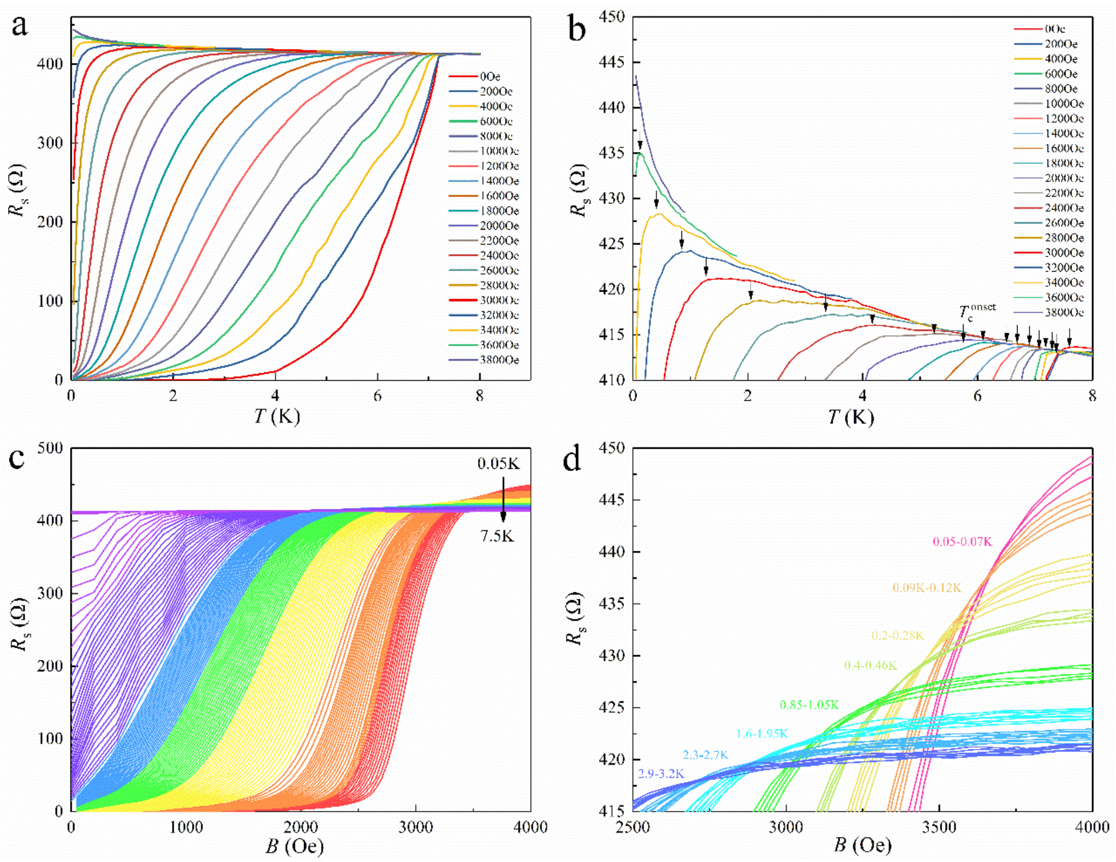

Figure S10. Transport properties of another graphene/Pb-islands-array hybrid. a) *R*-*T* curves measured under perpendicular magnetic field from 0 Oe to 3800 Oe. b) Enlarged view of the transition area in panel a. Black arrows indicate the onset temperature of the superconducting state. c) Magnetoresistance isotherms measured at different temperatures in 10 mK steps from 50 mK to 200 mK, 20 mK steps from 220 mK to 0.5 K, 50 mK steps from 0.55 K to 4 K and 0.1 K steps from 4.1 K to 7.5 K. d) Enlarged image of the multiple critical behavior region of panel c.

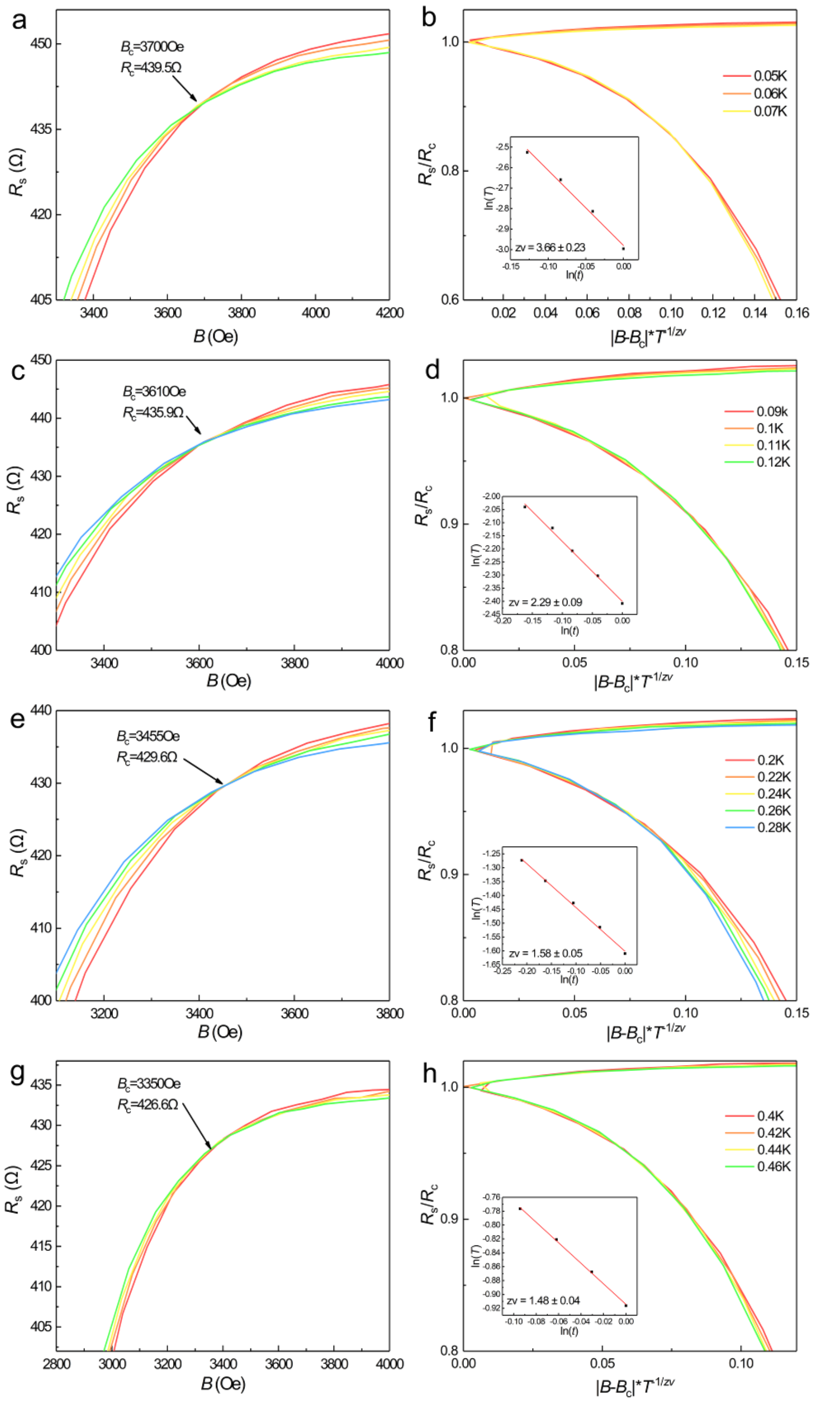

Figure S11. Finite-size scaling analysis for different "critical" points for another graphene/Pb-islands-array hybrid from 0.05 K to 0.46 K. a, c, e, g) $R_s(B)$ characteristics of different temperatures crossover at "critical" point (B_c, R_c). b, d, f, h) Normalized R_s as a function of the scaling variable $\left|B - B_c\right|(T / T_0)^{-1/zv}$. Inset shows the relationship between the temperature and the scaling parameter t as $t = T^{-1/zv}$ in a log-log scale plot and the critical exponent zv is derived from the linear fitting.

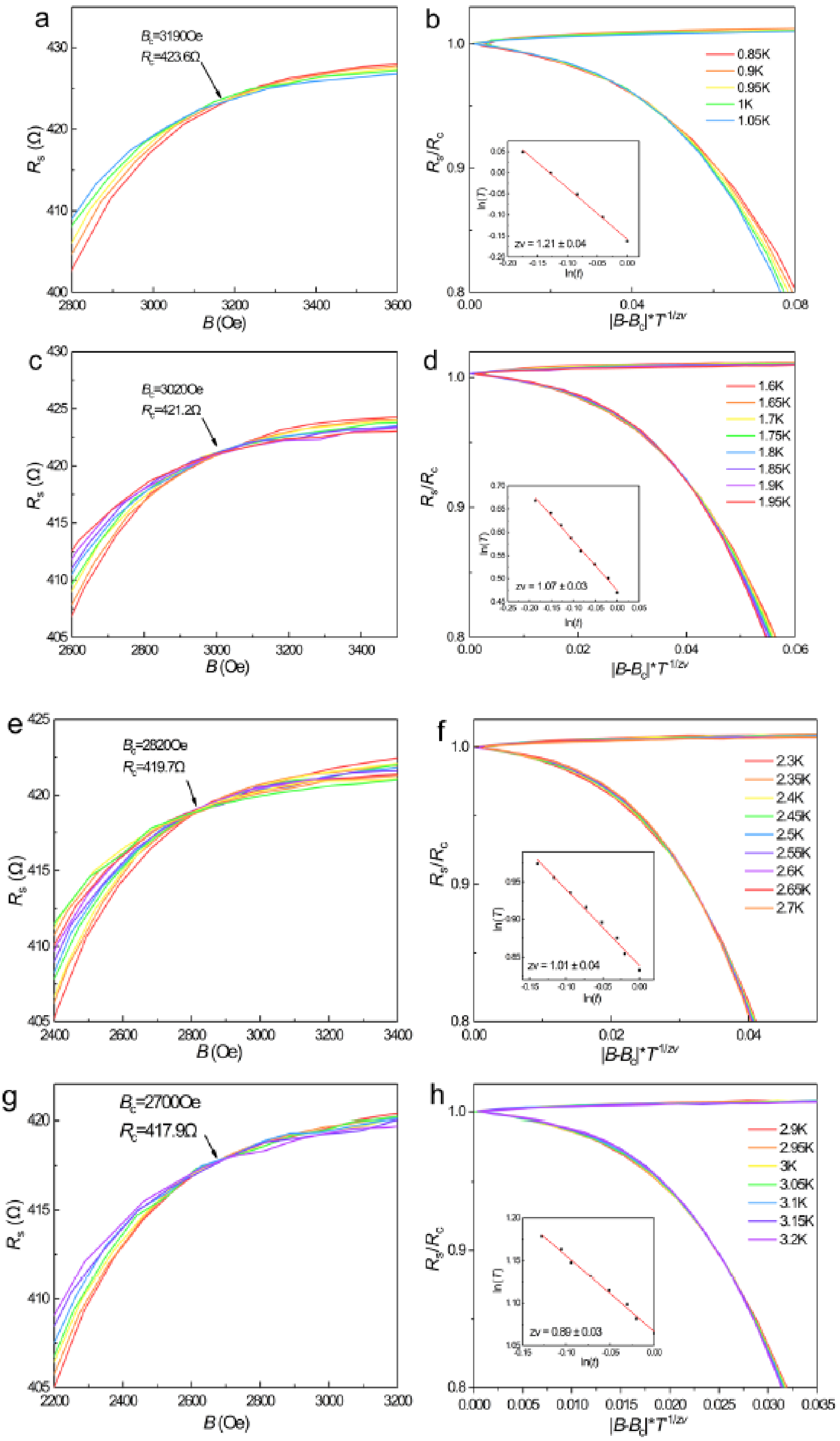

Figure S12. Finite-size scaling analysis for different "critical" points for another graphene/Pb-islands-array hybrid from 0.85 K to 3.2 K. a, c, e, g) $R_s(B)$ characteristics

of different temperatures crossover at "critical" point (B_c, R_c). b, d, f, h) Normalized R_s as a function of the scaling variable $|B-B_c|(T/T_0)^{-1/z\nu}$. Inset shows the relationship between the temperature and the scaling parameter t as $t = T^{-1/z\nu}$ in a log-log scale plot and the critical exponent $z\nu$ is derived from the linear fitting.

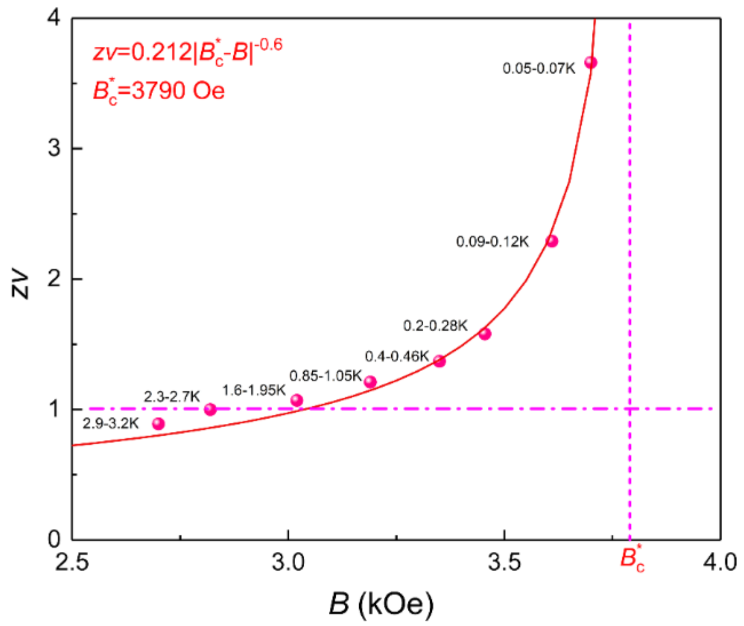

Figure S13. Exponent $z\nu$ as a function of magnetic field B for another graphene/Pb-islands-array hybrid. Exponent $z\nu$ values derived from FSS analysis increase rapidly when approaching the zero-temperature limit. The red solid line shows a fitting curve based on the activated scaling law shown in the upper left corner. Two dashed lines represent the constant values with B_c^* = 3790 Oe and $z\nu$ = 1.

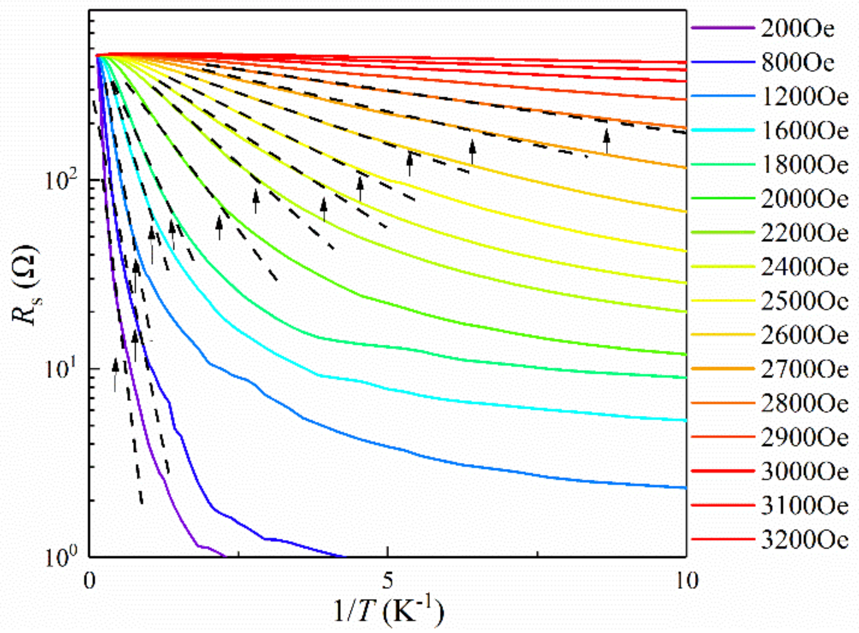

Figure S14. Arrhenius plot analysis of graphene/Pb-islands-array hybrid. Arrhenius plot of sheet resistance under different magnetic field shows the thermally activated flux flow region (fitted by the black dash line). The thermally activated temperature $T_{TAFF}(B)$ is defined as the temperature that deviates from the black dash fitting line, as denoted by the black arrow. The slope of the linear fit represents the activation energy.

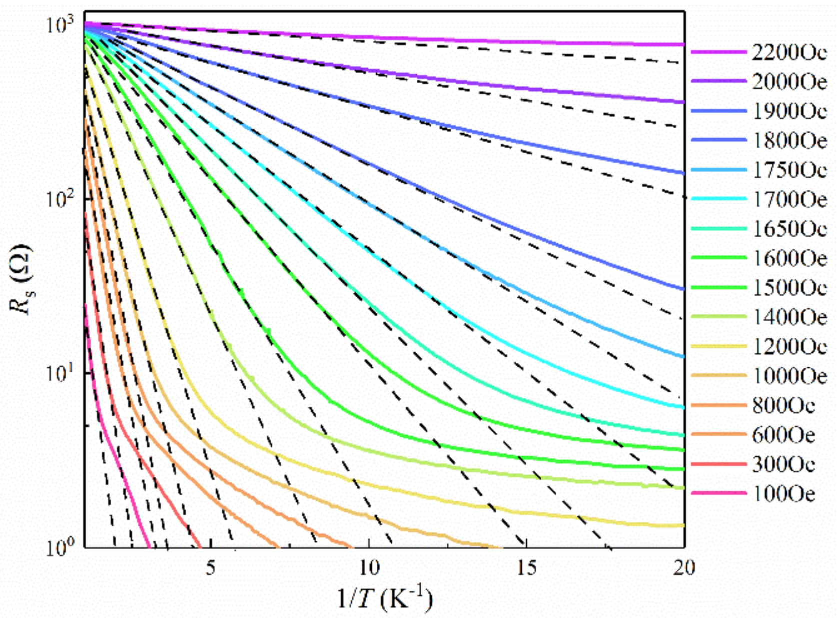

Figure S15. Arrhenius plot analysis of graphene/Sn-islands-array hybrid (data obtained from ref. 2 for a comparison). Arrhenius plot of sheet resistance under

different magnetic field shows the thermally activated flux flow region (fitted by the black dash line). The slope of the linear fit represents the activation energy.

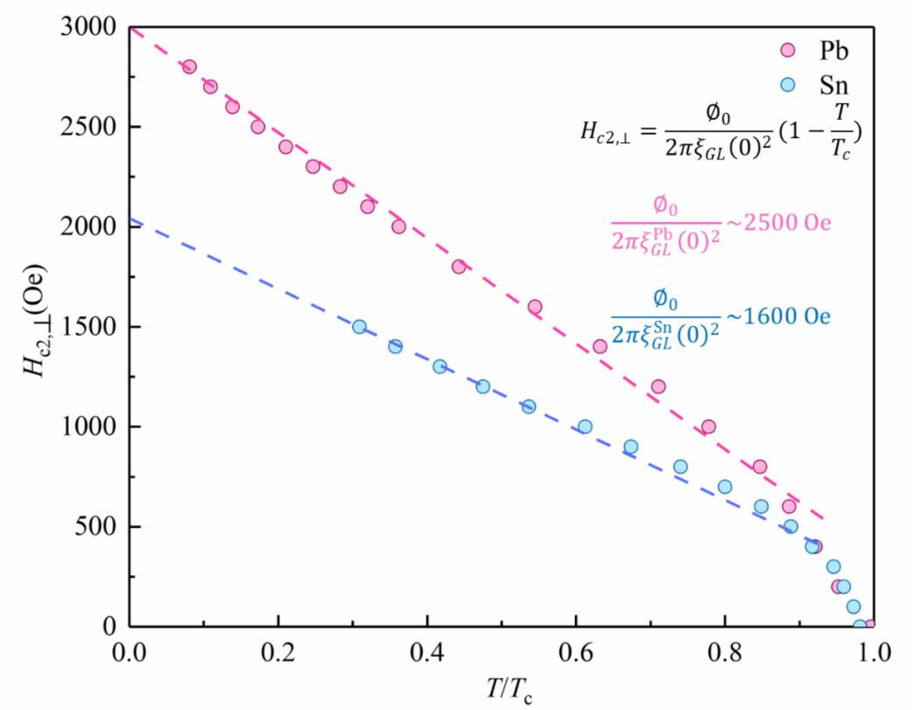

Figure S16 Extracted upper critical magnetic fields $H_{c2,\perp}$ defined as the 90% resistance of the normal state as a function of temperature. The pink dots extracted from graphene/Pb-islands-array hybrid in the main text and the blue dots extracted from graphene/Sn-islands-array hybrid [2]. The dash curves are fit using 2D Ginzburg-Landau equation expressed as $H_{c2,\perp} = \frac{\emptyset_0}{2\pi\xi_{GL}(0)^2}(1-\frac{T}{T_c})$, where $\xi_{GL}(0)$ is the zero-temperature GL in-plane coherence length and $\emptyset_0$ is the magnetic flux quantum.

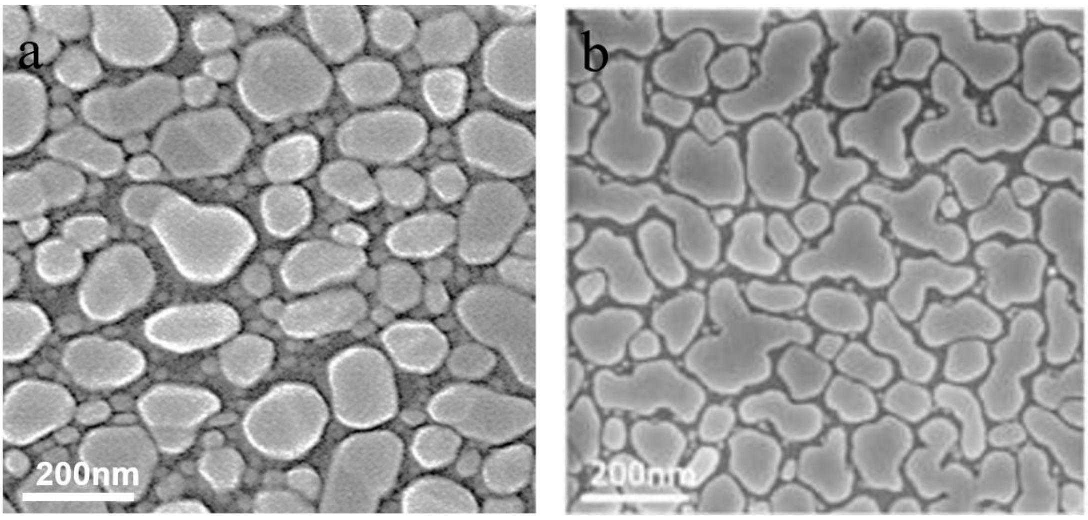

Figure S17. The comparison of the surface topographies. SEM images of graphene/Pb-islands-array hybrid (a) and graphene/Sn-islands-array hybrid (b) (image selected from ref. 2). In graphene/Pb-islands-array hybrid, there are considerable amounts of tiny scattering islands with only few nanometers surrounding the core islands, which rarely exist in graphene/Sn-islands-array hybrid.

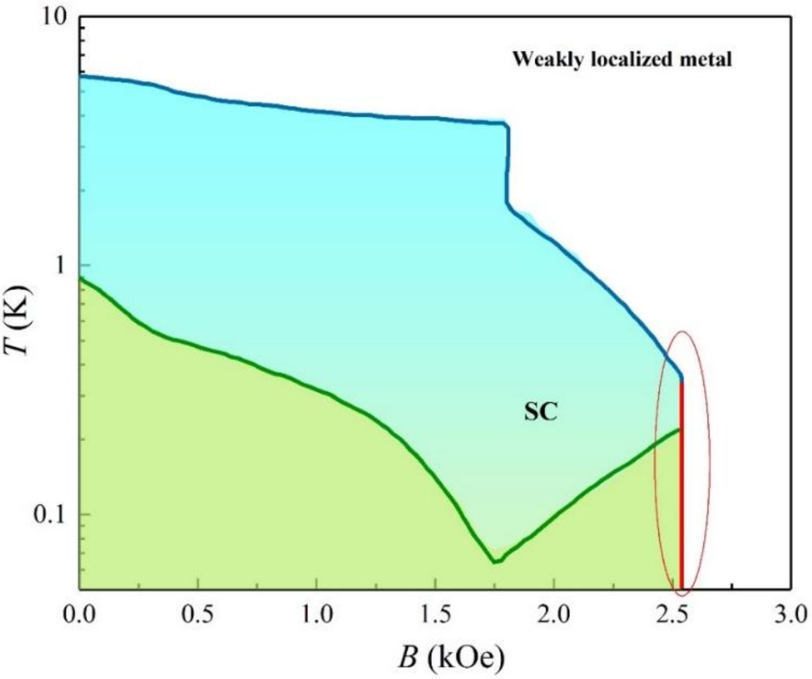

Figure S18. Phase diagram of graphene/Sn-islands-array hybrid (data collected from ref. 2). The superconducting dome exhibits a sharp transition at zero temperature as circled by the red line, which is quite different from our obversation in graphene/Pb-islands-array hybrid with quantum Griffiths singularity.

Supplementary References

1. Y. Saito, Y. Kasahara, J. T. Ye, Y. Iwasa, T. Nojima, *Science* **2015** 350, 409-413.
2. Y. B. Sun, H. Xiao, M. Zhang, Z. Y. Xue, Y. F. Mei, X. M. Xie, T. Hu, Z. F. Di, X. Wang, *Nat. Commun.* **2018** 9, 2159.
